# Acute mental change as the presenting sign of posthepatectomy hepatic failure: A case report

**DOI:** 10.1097/MD.0000000000018166

**Published:** 2019-11-27

**Authors:** Jin Young Chon, Hye Young Moon, Sangbin Han, Jueun Kwak, Ji Young Lee, Eun Sung Kim, Hyun Sik Chung

**Affiliations:** a Department of Anesthesiology and Pain Medicine, Yeouido St. Mary's Hospital; b Department of Anesthesiology and Pain Medicine, Seoul St. Mary's Hospital, College of Medicine, The Catholic University of Korea, Seoul, Republic of Korea.

**Keywords:** hepatectomy, hepatic failure, liver cirrhosis, liver function

## Abstract

**Rationale:**

Hepatectomy is a treatment to increase survival and curability of patients with intrahepatic lesions or malignant tumors. However, posthepatectomy liver failure (PHLF) can occur. This case is a patient showing acute mental change in postanesthetic care unit (PACU) as an uncommon symptom of PHLF after extended right hepatectomy.

**Patient concerns:**

A 68-year-old male patient was admitted for surgery of Klatskin tumor. He had hypertension and atrial fibrillation. His model for end-stage liver disease score was 16 pts. His serum bilirubin and ammonia levels were 4.75 mg/dL and 132.8 mcg/dL, respectively. Other laboratory data were nonspecific. He underwent extended right hepatic lobectomy including segments IV-VIII for 9 hours. Weight of liver specimen was 1028 g which was about 58% of total liver volume based on computed tomographic volumetry. The patient was extubated and moved to the PACU with stable vital sign and regular self-breathing. He could obey verbal commands. Fifteen minutes after admission to the PACU, the patient showed abruptly decreasing mental status and self-breathing.

**Diagnoses:**

Brain computed tomography, blood culture, and sputum culture were performed to diagnose brain lesions and sepsis for evaluating the sudden onset comatous mental status. Results showed nonspecific finding.

**Interventions:**

He was intubated for securing airway and applying ventilatory care. The patient was moved to the intensive care unit. He received intensive conservative therapy including continuous renal replacement therapy and broad-spectrum antibiotics.

**Outcomes:**

The patient's condition was worsened. He expired on postoperative day 3.

**Lessons:**

Acute mental change is uncommon and rare as initial symptoms of PHLF. Therefore, clinician may overlook the diagnosis of PHLF in patients with acute mental change after hepatectomy. Thus, clinician should plan an aggressive treatment for PHLF including liver transplantation by recognizing any suspicious symptom, although such symptom is rare.

## 1 Introduction

Hepatectomy for surgical treatment is indispensable for increasing patient survival and curability in the presence of intrahepatic lesions such as intractable lesions or malignant tumors.^[1]^ However, resection of the liver is limited by the anatomical structure of the liver. The most important factor of anatomical structure for liver resection is the anatomical structure of blood vessel of the liver. Sometimes it sometimes hepatic resection cannot be performed or extended resection including normal liver tissue is needed due to location of intrahepatic lesion and anatomical structure of the liver. The anatomical structure of the liver can be grossly divided according to the hepatic vein. Hepatectomy is conventionally performed along the left or right hepatic vein.^[2]^ If intrahepatic lesions invade beyond the area of left or right hepatic vein, an extended hepatectomy can be considered under limited situation of only involving near one segment of lesion's opposite side. Otherwise, resection of intrahepatic lesion cannot be performed. Extended hepatectomy usually permitted remnant liver volume (RLV) above 25% after hepatic resection to prevent posthepatectomy liver failure (PHLF). For these reasons, patients who require hepatectomy should be evaluated for pre-operative hepatic size or function to prevent PHLF. In particular, preoperative assessment of liver function is more important and significant if liver resection is planed more widely such as extended hepatectomy.

We present a case of acute uncommon mental change immediately posthepatectomy in postanesthetic care unit (PACU) as a symptom of acute hepatic failure after right extended hepatectomy due to Klatskin tumor in a patient with liver cirrhosis.

This case presentation and waiver of informed consent were approved by the Institutional Review Board of our hospital (KC19ZESI0011).

## 2 Case presentation

A 68-year-old male patient was admitted to our hospital with a chief complain of a jaundice that occurred a week ago. He has been diagnosed with Klatskin tumor by evaluation for a jaundice and scheduled for hepatic resection to remove the tumor. The plan of operation was right hepatectomy or extended right hepatectomy depending on surgical operation field. The patient had a diagnosis and medication for hypertension, cardiac arrhythmia of atrial fibrillation, and vascular dementia. These underlying diseases were well under control. There were no specific findings on preoperative echocardiography or pulmonary function test. Laboratory data were international normalized ratio (INR) of 1.35, serum ammonia level of 132.8 mcg/dL, and model for end-stage liver disease (MELD) score of 16 pts. Serum total bilirubin level was 33.39 mg/dL at admission and 4.75 mg/dL before operation. Serum total bilirubin level was controlled by percutaneous transhepatic biliary drainage under diagnosis of biliary stasis due to hilar cholangiocarcinoma. Other serum electrolytes including creatinine were within normal range. To estimate RLV, liver computed tomographic volumetry was done preoperatively. Results were: total volume, 1761.49 cm^3^; left lobe, 668.94 cm^3^; right lobe, 1055 cm^3^; left lateral segment, 278.85 cm^3^; and caudate lobe, 37.55 cm^3^. RLV was predicted to be about 40.1% which was a tolerable RLV after hepatectomy.

In operation room, his systolic/diastolic blood pressure was measured at 160/100 mm Hg. His heart rate was 97 beats per minute with irregular rhythm due to atrial fibrillation. For induction of anesthesia, 2 mg/kg of propofol and 0.6 mg/kg of rocuronium were used. After intubation, balanced anesthesia was done using by 5 to 6 vol% of desflurane with 40% of FiO_2_ mixed to medical air and continuous infusion of 0.05 to 0.1 mcg/kg/min of remifentanil. Left radical arterial cannulation was done using 20 gauge angio-catheter for monitoring continuous blood pressure and measuring laboratory tests. The central venous catheter was placed in the right subclavian vein for the administration of fluid and adrenaline drugs. Right hepatic lobectomy was done including segments IV-VIII at 4 hours 30 minutes after starting the operation. The weight of liver specimen was 1028 g. Total operation time was 9 hours. Blood loss volume was about 1200 mL. Two packs of packed red blood cells and 2 packs of fresh frozen plasma were transfused. At the end of operation, the patient was extubated successfully with stable hemodynamic vital sign, and regular spontaneous breathing. The patient also showed recovered mental status. He could obey verbal commands. After confirming that the patient's vital sign and mental status were recovered to almost the same as the preoperative status, the patient was transferred to PACU.

The patient had an appropriate verbal and motor response to verbal commends such as saying one's name and opening one's mouth by verbal command at arrival of PACU. However, 15 minutes after admission to the PACU, the patient's consciousness became worse abruptly. He showed decreased response to painful stimuli. Peripheral pulse oximetry was decreasing below 80%. We applied Ambu-bag to overcome respiratory arrest. However, it did not work. We performed a tracheal intubation and started intermittent positive pressure ventilation because Ambu-bag respiration assist was ineffective. At that time, arterial blood gas analysis showed pH of 7.32 and lactate level of 47.57 mg/dL suitable for diagnosis of lactic acidosis. Brain computed tomography (CT) was immediately performed to distinguish the cause of acute loss of consciousness arising new brain lesions such as ischemia or intracranial hemorrhage because the patient had vascular dementia. However, brain CT showed no specific findings.

**Figure 1:**
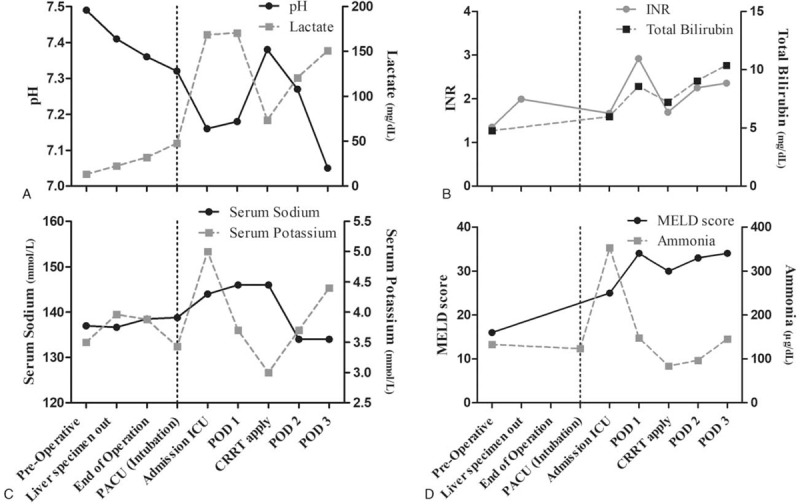
Time course of laboratory values and MELD score. (A) Serum pH and lactate, (B) INR and total bilirubin, (C) serum sodium and potassium, (D) MELD score. CRRT = continuous renal replacement therapy, ICU = intensive care unit, INR = international normalized ratio, MELD = model for end-stage liver disease, PACU = post-anesthetic care unit, POD = postoperative day.

The patient was transferred to intensive care unit (ICU). After 4 hours of admission at the ICU, unstable vital sign was observed despite the use of vasopressors. Physician treated the patient under the impression of septic shock caused by infected bile juice. Samples of blood and sputum were sent to the laboratory for evaluating sepsis. On postoperative day (POD) 1, sustained hypotension continued despite administration of vasopressors. Furthermore, metabolic acidosis continued and urine was decreased due to acute renal failure (ARF). Continuous renal replacement therapy was applied to overcome sustained metabolic acidosis and ARF. On POD 3, blood and sputum results were reported to be negative. Despite persistent and intensive supportive therapies, hypotension, metabolic acidosis, and comatous mentality continued and got worse. Eventually, the patient expired on POD 3. Figure 1 shows laboratory results over time.

## 3 Discussion and conclusions

We report a rare case of acute mental change as a symptom of acute hepatic failure immediately after extended right hepatectomy in a patient with liver cirrhosis. The incidence of PHLF has been reported to be up to 8%.^[3]^ Risk factors of PHLF include pre-existing liver disease, male gender, advanced age (>65 years), small RVL (≤25%), excessive blood loss (>1250 mL), and prolonged operation time.^[4]^ The patient of our report had risk factors of PHLF such as liver cirrhosis, advanced age, excessive blood loss, and prolonged operating time. Therefore, the patient was highly possible to develop PHLF despite estimated RVL >25%. Scoring systems reflecting liver function in patients with cirrhosis for accessing feasibility of liver resection include the Child-Pugh score and MELD score. With respect to MELD score, MELD >11 pts could predict a PHLF.^[5,6]^ The MELD score of the patient in the present case was 16 at the time immediately before surgery. Therefore, preoperative laboratory results of the patient also could predict the possibility of PHLF even if the estimated RVL was above 25% based on CT volumetry.

Manifestation of PHLF is similar to systemic inflammatory response such as sepsis and multiple organ dysfunction syndrome (MODS). However, acute mental change as initial symptom of PHLF is rare. Typical interval of the onset of encephalopathy is 1 to 2 weeks. It can be up to 6 months after hepatic failure.^[7]^ The following could be considered as reasons for unusual manifestation of symptom. PHLF of this case was developed abruptly due to sudden large loss of liver mass and function by surgical removal. Thus, there was little time to influence the synthesis of coagulation factors which was manifested by the prolonged INR and activated partial thromboplastin time commonly seen in PHLF after loss of liver function. The loss of consciousness appeared as an initial symptom of PHLF in this case and it might have been more serious and advanced because it was accompanied by sudden acidosis that caused respiratory acidosis as well as metabolic and lactic acidosis. We confirmed that the cause of the patient's acute mental change was not a result of brain lesions, such as ischemia and hemorrhage, by performing a brain CT at the time of acute mental change in the PACU. Therefore, the cause of the patient's acute mental change could be assumed to be due to either a systemic inflammatory response, such as sepsis, or PHLF and eventually, PHLF was confirmed as the cause of the acute mental change. Negative blood and sputum culture results were, unfortunately, reported on the day the patient expired.

With respect to the brain imaging study, the brain CT findings, including brain edema, were nonspecific in the context of the patient's comatose mental status. In previous report, only half of the patients with grade 3 to 4 hepatic encephalopathy showed brain edema in brain CT images.^[8]^ Thus, it is not surprising that the brain CT finding was non-specific in a patient with acute mental change resulting from PHLF, as in this case. The potential pathway of rapid progressive mental change due to PHLF is hyperammonemia in the serum. Hyperammonemia can cause neuronal dysfunction by decreasing excitatory neurotransmission in the brain and leads to hepatic encephalopathy, in which shows mental change is a symptom.^[9]^ The patient's serum ammonia was 352.8 mcg/dL at time of admission to the ICU. It was enough to cause ammonia-induced neurotoxicity through the blood-brain barrier.

Initial treatment was performed using supportive treatment and broad-spectrum antibiotics including performing blood culture regarding impression of sepsis with proceeding MODS. Physician considered the cause of mental change was sepsis due to ascending infection on sustained bile juice by hilar cholangiocarcinoma. However, blood culture showed negative findings on the final report at 3 days after incubation. The patient expired even though treatment for hepatic failure was initiated after exclusion of sepsis.

Estimation of posthepatectomy residual liver function (PHRLF) plays an important role in preventing PHLF. CT volumetry is one of precious methods for estimating PHRLF.^[10]^ CT volumetry was also used for the patient in our case to estimate PHRLF. Results showed that the PHRLF was enough to prevent PHLF. Volumetric data showed 61% of RLV after hepatic resection which was a favorable result for resecting liver parenchyma because the estimated volume of resection liver was below 50% of whole liver volume.^[11–13]^ However, the patient was under a condition of LC. The result of CT volumetry for preventing PHLF must be guaranteed limited situation of functionally normal liver. Therefore, the method of estimating PHRLF was inappropriate in our case. Other tests of PHRLF should be performed to predict and prevent PHLF. Other methods of estimating PHRLF include levels of total bilirubin and indocyanin green (ICG)-15 clearance test. If there is an obvious situation of predicting PHLF, physician should consider modified surgery such as staged hepatectomy or hepatectomy after portal vein embolization to prevent PHLF. Surgical resection should be attempted for hepatocarcinoma rather than canceling the operation to minimize risk of the patient.

In conclusion, it is important to require more dedicated methods for estimating PHLF in patients with liver disease that may interfere with liver function such as LC. Although volumetric data can quantity the liver, they do not represent the quality or function of the liver. Therefore, additional functional tests for RLF should be performed to prevent PHLF in patients with existing liver disease. In addition, clinician should provide aggressive treatment for PHLF including liver transplantation by recognizing any suspicious symptom related to PHLF, including acute mental change, although it is a rare symptom of initial PHLF.

## Author contributions

**Conceptualization:** Hyun Sik Chung.

**Data curation:** Hye Young Moon, Sangbin Han, Jueun Kwak.

**Investigation:** Jin Young Chon.

**Project administration:** Hyun Sik Chung.

**Resources:** Hye Young Moon, Sangbin Han, Jueun Kwak, Ji Young Lee.

**Software:** Sangbin Han, Ji Young Lee, Eun Sung Kim.

**Supervision:** Hyun Sik Chung.

**Validation:** Ji Young Lee, Eun Sung Kim.

**Writing – original draft:** Jin Young Chon.

**Writing – review and editing:** Hyun Sik Chung.

Jin Young Chon orcid: 0000-0002-9827-5883.

Hyun Sik Chung orcid: 0000-0001-7527-1866.
